# Development of Novel Vaccines against Enterovirus-71

**DOI:** 10.3390/v8010001

**Published:** 2015-12-30

**Authors:** Pinn Tsin Isabel Yee, Chit Laa Poh

**Affiliations:** Virology Research Group, Vice Chancellor’s Office, Sunway University, Bandar Sunway, Kuala Lumpur, Selangor 47500, Malaysia; isabely@sunway.edu.my

**Keywords:** Enterovirus 71, hand, foot and mouth disease, inactivated vaccine, viral like particles

## Abstract

The hand, foot and mouth disease is caused by a group of Enteroviruses such as Enterovirus 71 (EV-A71) and Coxsackievirus CV-A5, CV-A8, and CV-A16. Mild symptoms of EV-A71 infection in children range from high fever, vomiting, rashes and ulcers in mouth but can produce more severe symptoms such as brainstem and cerebellar encephalitis, leading up to cardiopulmonary failure and death. The lack of vaccines and antiviral drugs against EV-A71 highlights the urgency of developing preventive and treatment agents against EV-A71 to prevent further fatalities. Research groups have developed experimental inactivated vaccines, recombinant Viral Protein 1 (VP1) vaccine and virus-like particles (VLPs). The inactivated EV-A71 vaccine is considered the safest viral vaccine, as there will be no reversion to the infectious wild type strain. The recombinant VP1 vaccine is a cost-effective immunogen, while VLPs contain an arrangement of epitopes that can elicit neutralizing antibodies against the virus. As each type of vaccine has its advantages and disadvantages, increased studies are required in the development of such vaccines, whereby high efficacy, long-lasting immunity, minimal risk to those vaccinated, safe and easy production, low cost, dispensing the need for refrigeration and convenient delivery are the major goals in their design.

## 1. Introduction

Vaccination for various viral diseases has markedly reduced mortality and morbidity worldwide for more than 200 years. Indeed, the greatest public health success can be attributed to vaccination. Nevertheless, the future is abound with challenges as there remains many diseases that do not yet have effective vaccines against Human Immunodeficiency virus/Acquired Immunodeficiency Syndrome (HIV/AIDS), Dengue virus, Ebola, Hepatitis C, and the Enterovirus 71 which causes hand, foot and mouth disease. Every year, two new species of viruses are added to the list of approximately 200 different infectious viruses [1]. In addition, some viruses, particularly RNA viruses, can emerge as new pathogens as they have high mutation rates due to their error-prone RNA dependent RNA polymerase. The existing viruses can evolve to become more virulent through recombinations or mutations and the mutated strains often do not have any effective vaccines against them.

Currently, there are only an estimated 15 vaccines to combat the 200 viruses known to infect man [1]. Although there are more vaccines undergoing clinical trials, it is worrying to note that humans remain vulnerable to the existing 180 or so viruses that have no effective vaccines. Therefore, increased research is required to develop new and better vaccines, whereby high efficacy, long-lasting immunity, minimal risk to those vaccinated, safe and easy production, low cost, dispensing the need for refrigeration and convenient delivery are the major goals in their design.

## 2. Enterovirus 71

The World Health Organization (WHO) and the scientific community have been addressing challenges unprecedented in public health posed by Enteroviruses in the post-poliovirus era. Enteroviruses such as Enterovirus 71 (EV-A71), Coxsackie type A16 (CV-A16) and other enteroviruses causing hand, foot and mouth disease (HFMD) have led to over seven million infections, including 2457 fatalities in China from 2008 to 2012 [2]. However, due to increasing travels and rapid globalization, outbreaks in other parts of the world have also appeared in other regions in cyclical epidemics [3].

Table 1 summarizes the various clinical symptoms associated with enteroviral infections. Within the family *Picornaviridae*, the genus *Enterovirus* comprises 12 species. The species Enterovirus A consists of 25 serotypes and includes the enteroviruses causing HFMD such as EV-A71, CV-A16, CV-A5, CV-A6, CV-A8 and CV-A10 [4]. Serotypes such as CV-A4 and CV-A5 are more often associated with herpangina. Five years of virological surveillance in China (2008–2014) showed that 43.73%, 22.04%, and 34.22% of HFMD cases were due to EV-A71, CV-A16 and other enteroviruses, respectively [5].

**Table 1:** Clinical manifestations of enteroviruses.

Enterovirus Serotypes	Clinical Manifestations
Poliovirus 1 to 3 Echovirus 4, 6, 9, 11, 30; Enterovirus 71	Paralysis
Poliovirus 1 to 3; Coxsackievirus A2, A4, A7, A9, A10, B1 to B6; Echovirus 1 to 11, 13 to 23, 25, 27, 28, 30, 31; Enterovirus 70, 71	Aseptic meningitis
Coxsackievirus A5, A8, A10, A16, Enterovirus 71	Hand, foot and mouth disease (HFMD)
Coxsackievirus A2 to A6, A8, A10	Herpangina
Coxsackievirus A24, Enterovirus 70	Acute hemorrhagic conjunctivitis
Echovirus 2, 6, 9, 19	Encephalitis
Coxsackievirus B1 to B5, Enterovirus 71	Meningoencephalitis
Coxsackievirus B3	Pericarditis, myocarditis

Each symptom may potentially be caused by more than one enterovirus [6].

However, *Coxsackie* viruses which are common etiological agents of HMFD do not generally cause neurological or cardiopulmonary disease and EV-A71 is the main causative agent of fatal HFMD infections [7]. In major outbreaks, EV-A71 could contribute to 80%–85% of HFMD-related deaths as observed during the HFMD outbreak in Taiwan in 1998 [8]. In an epidemiological survey in Shanghai from May 2010 to April 2011, it was reported that 83.8% of HFMD cases were due to EV-A71, 9% to CV-A10, 8.3% to CV-A6 and CV-A16 accounted for 6.9% of total HFMD cases [9].

**Figure 1:**
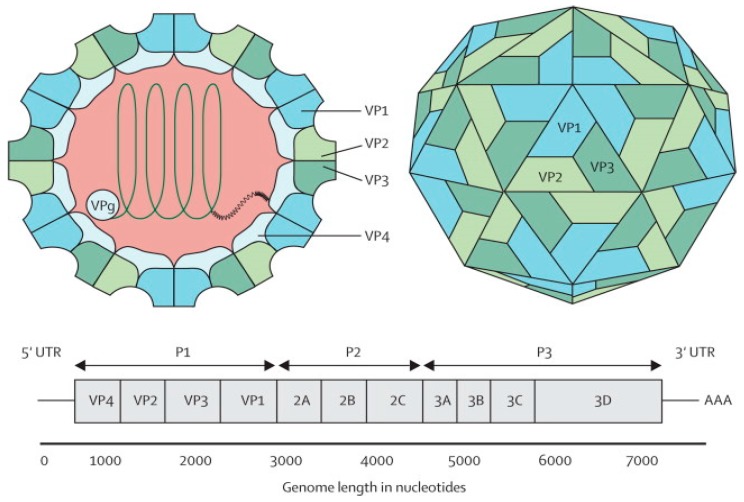
Structure and genome of Enterovirus 71. The capsid consists of 60 protomers, each consisting of four polypeptides that comprise the structural proteins: VP1, VP2, VP3, and VP4 and are encoded by the P1 region of the genome. The P2 and P3 regions encode for seven non-structural proteins: 2A–2C and 3A–3D (the EV-A71 genome is represented by the green line, followed by poly-A residues at the 3′UTR). Reproduced from ViralZone, with permission from Swiss Institute of Bioinformatics [12].

The EV-A71 virus is classified as a Human enterovirus A (HEV-A) species, belonging to the genus *Enterovirus* in the family *Picornaviridae*, together with some Coxsackie A viruses [10]. Phylogenetic analysis suggests that EV-A71 originated from CV-A16 from as early as 1941 [11]. The EV-A71 virus is a non-enveloped icosahedral viral particle that contains a single-stranded, positive sense, polyadenylated viral Ribonucleic Acid (RNA) of approximately 7.4 kb (Figure 1). The capsid is made up of 60 protomers, each consisting of 4 polypeptides that comprise the structural proteins, VP1, VP2, VP3 and VP4. Of all the polypeptides, VP4 is located on the internal side of the capsid while VP1, VP2 and VP3 are located on the external surface of the EV-A71 virus [12].

The EV-A71 genome comprises a 5′ non-translated region (5′NTR), a long open reading frame (ORF) and a short 3′NTR followed by a polyadenylated (poly A) tail. The 5′NTR contains an internal ribosome entry site (IRES) which allows viral protein translation in a cap-independent manner [13]. The ORF is translated into a single large polyprotein of approximately 2100 amino acids, which is divided into three regions (P1–P3). The polyprotein undergoes a series of processing events, culminating in the maturation cleavage of the polyprotein, which generates structural and non-structural viral proteins [14,15]. The four structural proteins, VP1, VP2, VP3 and VP4, are encoded by the P1 region, which constitutes the virus capsid. Proteins derived from the non-structural P2 (2Apro, 2B, 2BC, and 2CATPase) and P3 (3A, 3AB, 3B, 3Cpro, 3CDpro, and 3Dpol) regions are most directly involved in virus replication, structural and biochemical changes which are observed within the infected cell [15]. Non-structural proteins (2A and 3C proteinases) are responsible for apoptosis of infected cells *in vitro* [16,17].

**Figure 2:**
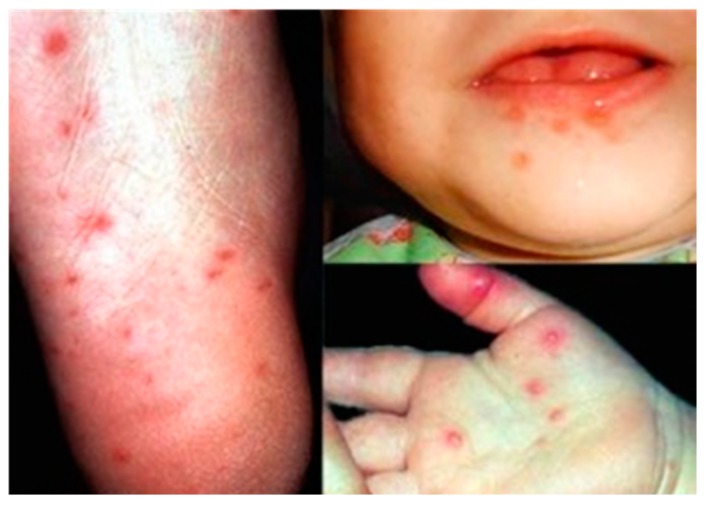
Vesicles on the foot, mouth and palm area of children infected with hand, foot and mouth disease (HFMD). Adapted from the Dermatologic Image Database, Department of Dermatology, University of Iowa College of Medicine, USA, 1996 (http://tray.dermatology.uiowa.edu/ImageBase). (Permission granted by University of Iowa) [19].

The EV-A71 virus commonly causes the hand, foot and mouth disease (HFMD) in young children less than 6 years of age. Although EV-A71 started circulating as early as 1963 in the Netherlands, EV-A71 was first reported to be isolated in 1969 from the stool specimen of an infant with serious nervous system disease in California [18]. Mild symptoms of EV-A71 infection in children range from fever (≥39 °C), sore throat, loss of appetite and rash with vesicles on hands, foot and diaper area. In addition, rupture of the vesicles would lead to ulcers in the throat, mouth and tongue (Figure 2).

EV-A71 can produce more severe symptoms such as aseptic meningitis, brain stem encephalitis, acute flaccid paralysis, neurogenic pulmonary edema, delayed neurodevelopment and reduced cognitive function [7]. In 1997, an outbreak of EV-A71 caused 41 deaths in Sarawak, Malaysia. This was followed by a large outbreak in Taiwan involving over 100,000 cases, which led to 78 fatalities [20]. In more recent years, large outbreaks with high fatalities occurred across the Asia Pacific in countries like China, Cambodia and Vietnam [21]. For example, 54 out of the 78 HFMD cases in a 2012 outbreak in Cambodia were highly fatal. 

Enteroviruses such as EV-A71 and CV-A16 causing HFMD have led to over 10 million infections, including 3046 fatalities in China from 2008 to June 2014 [2]. In Vietnam, there were 13 deaths out of 49,317 cases of infection [22]. Some of these young children died of complications due to pulmonary edema, while others could not survive brain and spinal cord inflammations due to virulent genotypes of EV-A71 [23]. The lack of vaccines and antiviral drugs against EV-A71 highlights the urgency and significance of developing preventive and treatment agents against EV-A71 to prevent further fatalities.

## 3. Potential Candidates for EV-A71 Vaccine

There is considerable interest in the development of EV-A71 vaccines as HFMD has become a severe global and life-threatening disease in infants and young children. In 2014, China reported over 2.7 million cases of HFMD caused by EV-A71, CV-A16, and other enteroviruses, that led to approximately 243 deaths [22]. Research groups have developed experimental inactivated vaccines [24], recombinant VP1 vaccine [25], live attenuated vaccines [26,27], virus-like particles [28], synthetic peptide vaccine [29,30] and DNA vaccine [31].

Each type of vaccine has its own advantages and disadvantages. The inactivated EV-A71 vaccine is considered the safest viral vaccine as there will be no reversion to the infectious wild type strain. Reversion of the live attenuated poliovirus vaccine strains generally happens in 1 out of 750,000 people vaccinated, but this occurrence will not happen with the inactivated poliovirus vaccine. Zhu *et al*. (2014) [32] showed that the inactivated EV-A71 vaccine was highly immunogenic and elicited antibodies in children with a neutralizing titer of 1:16 that provided protection against mild to severe HFMD for at least 1 year in children.

Nevertheless, the inactivated vaccine has several major disadvantages as immunogenicity is not long-lasting and requires multiple boosters. This is because inactivated vaccines only initiate the humoral immunity and lacks cellular immunity (CD8^+^ T cells) responses. In an inactivated vaccine, there is no viral replication and hence, lower antigen content and less prolonged antigen persistence is expected [33]. As a result, inactivated vaccines elicit weaker and shorter antibody responses and no long-term immune memory. This explains the need for multiple boosters after administration of inactivated vaccines. Another disadvantage of the EV-A71 inactivated vaccine would be the failure to prevent CV-A16 infections and this could compromise the acceptability of the inactivated monovalent EV-A71 vaccine. The study of Chong *et al.* (2015) [34] also concurred with the results that even though the efficacy of inactivated EV-A71 vaccine was more than 90% against EV-A71-related HFMD, only around 80% protection against EV-71 associated serious diseases was reported. The vaccine did not protect against CV-A16 infections either.

Increasingly, there has been more research to develop recombinant VP1 vaccines that contain VP1 as the major neutralizing antigen, harnessed into vaccine vectors. They are advantageous as they are cost-effective immunogens when compared to the inactivated vaccines and are safe. This was confirmed by Chen *et al.* (2006) [35] who showed that when transgenic tomatoes expressing the VP1 protein were fed to Balb/c mice as an oral vaccine, the serum from the immunized mice showed IgG and IgA neutralizing titers of 1:16. The vaccine had elicited *both* humoral and cellular immune response from the mice. The serum was also able to neutralize EV-A71 infection in Rhabdomyosarcoma cells. This demonstrates the potential of recombinant VP1 vaccines as good vaccine candidates.

However, recombinant VP1 vaccines produced lower levels of NtAb in vacinees. This increases the risk of unwanted immune responses such as antibody-dependent enhancement (ADE). ADE is a phenomenon whereby pre-existing sub-neutralizing antibodies could not inhibit virus entry and replication. ADE has been observed for EV-A71 [36], poliovirus [37], and CVB [38]. Han and colleagues (2011) [36] demonstrated that previous exposure to an avirulent strain of EV-A71 before another EV-A71 infection increased the risk factor for developing severe neurological complications and deaths. They also concluded that the presence of sub-neutralizing levels of antibodies exacerbated EV-A71 infection. The possible impact of ADE has to be taken into account when designing vaccines to prevent unwanted induction of enhancing antibodies. Therefore, a better alternative would be to use live attenuated vaccines that elicit both humoral and cellular immune responses if genetically stable, non-revertible EV-A71 vaccine strains could be constructed.

## 4. Inactivated EV-A71 Vaccines

Up to date, five organizations have completed pre-clinical studies to develop an inactivated vaccine which is at different phases of clinical trials. Three of the companies are from mainland China while the other two are from Taiwan and Singapore, respectively. The three China-based biopharmaceutical companies are Vigoo, Sinovac and Chinese Academy of Medical Science (CAMS). They have all completed Phase III Clinical Trials in 2014 for an inactivated EV-A71 vaccine against the sub-genotype C4 as it was the main sub-genotype responsible for outbreaks in China (Table 2). The three companies conducted randomized, double-blind, placebo-controlled, multicenter trials involving over 30,000 healthy children. Each candidate received two intramuscular doses of vaccine or placebo within a span of 28 days apart [39].

**Table 2:** Five EV-A71 inactivated vaccines at different clinical phases of development.

Organizations	Cell Lines and EV-A71 Strain	Clinical Trials Dosage (µg of EV-A71 Antigen)	Population Target	Current Status of Clinical Trial	Adjuvant	Technology for Vaccine Production
NHRI (Taiwan)	Vero cell and EV-A71 B4 (GMP-certified)	5 and 10	Young adults	Phase 1 completed	Aluminum phosphate	Roller bottles
Sinovac (China)	Vero cell and EV-A71 C4	1	Young adults, young children and infants	Phase 3 completed 1, 2 and 3 completed	Aluminum hydroxide	Cell factory
Beijing Vigoo (China)	Vero cell and EV-A71 C4	0.8	Young adults, young children and infants	Phase 3 completed Phase 1, 2 and 3 completed	Aluminum hydroxide	Microcarrier bioreactors, fermentation cylinder
CAMS (China)	Human diploid cell KMB-17 and EV-A71 C4	0.25	Young adults, young children and infants	Phase 3 completed 1, 2 and 3 completed	Aluminum hydroxide, glycine	Microcarrier bioreactors
Inviragen (Singapore)	Vero cell and EV-A71 B3	0.3 and 3	Young adults	Phase 1 completed Phase 1 Completed	Aluminum hydroxide	Cell factory

Sinovac reported that their inactivated vaccine efficacy was 94.8% and anti-EV-A71 immune response elicited by the two dose vaccines were found in 98.8% of participants. In addition, the anti-EV-A71 neutralizing titre of 1:16 associated with protection against EV-A71 was expected to last for at least one year. However, the neutralizing antibody (NtAb) titer was found to decline by 50% after six months [40]. In addition, there were different NtAb levels induced by the three vaccine strains although they were all from the sub-genotype C4a strain. Production of inactivated vaccines involves growing large amounts of the pathogenic EV-A71 C4 genotype strain in large cultivating vessels. Workers could be exposed to significant risks due to the pathogenic strain being used and to the formaldehyde which is a carcinogen. Additional processing steps such as gel filtration and/or ion-exchange chromatography would also incur significant cost.

The NtAb levels (Geometric Mean Titers, GMT) in children vaccinated with the inactivated vaccines produced by Sinovac and CAMS were 191 and 170 at one year post-vaccination and four weeks, respectively. The lowest VNA GMT was observed with the inactivated EV-A71 vaccine from Vigoo at a GMT of 92 at one year post-vaccination. This could be due to the different manufacturing processes, cell substrates, culture systems and vaccine doses being used by the three companies [34]. Mao *et al.* (2012) [41] also discovered that the aluminum hydroxide adjuvant used, though similar in concentration, had differing immunological-enhancing effects. Compared with the vaccine strains without the adjuvant, the differences in immunogenicity among the vaccine strains absorbed with alum adjuvant produced by the three manufacturers were increased, especially at 14 and 28 days after immunization.

The inactivated vaccines containing aluminum adjuvants when used at the lowest dose (162U) showed good protective effects in suckling mice against lethal challenge (90%–100% survival). 

The inactivated vaccines should be produced based on international manufacturing process criteria, global vaccine standards and high regulation of quality. Currently, roller bottles and cell factories are employed for upstream cell culture but they are labor intensive. Chong *et al.* (2012) [42] reported the feasibility of producing a C4-based inactivated vaccine (0.25 µg) at US $0.1/dose using a 40-L pilot scale batch reactor. Upstream manufacturing processes could be further improved with the use of bioreactors, micro-carriers and perfusion technology. To lower the production cost, a simple and efficient downstream chromatographic purification step will need to be incorporated. To determine the potency and efficacy of inactivated vaccines produced by different manufacturing processes, there is a need for standardization of the vaccine strain, quality control reagents, immunoassays and animal models at the international level, probably coordinated by WHO to achieve similar results like the Global Eradication Program for Polio. A global surveillance network for enterovirus outbreaks is needed to monitor immune responses to the inactivated EV-A71 vaccine.

Prior to the release of the inactivated EV-A71 vaccine, there were a few studies that are addressing whether the NtAb elicited by one EV-A71 sub-genotype could cross-neutralize other sub-genotypes or confer protection across genotypes or sub-genotypes. For example, it was reported that neutralizing antibodies elicited by 10 strains of the C4 genotype in rabbits had variable cross-neutralizing effects against different strains of the same sub-genotype and the genotype A BrCr strain, while another study demonstrated that mice challenged with lethal doses of B3 genotype survived due to prior vaccination with a C4 genotype vaccine [43]. In addition, Zhang *et al.* (2014) [44] showed that the titers of NtAb in children elicited by the EV-A71-C4a vaccine were higher against EV-A71-B4, B5, C1, C2 and C4b than against other EV-A71 sub-genotypes such as the A genotype. Interestingly, the NtAb titers raised against the C4a sub-genotype used for immunization was even lower than those of all the other EV-A71 sub-genotypes and this indicated that the current genotyping scheme may not truly reflect their antigenicity.

It is important to conduct studies with more antisera raised against the inactivated vaccine collected from future human phase III clinical trials to further evaluate the efficacy of cross-protection against all EV-A71 genotypes and sub-genotypes, determine the types of immune response and understand the immune correlates of protection. There is no data to show vaccine efficacy against serious EV-A71-associated neurologic disease, such data might become available after the vaccines are licensed and post marketing surveillance is undertaken. A global surveillance network to monitor the emergence of new EV-A71 strains after the introduction of the vaccine should be established. As the child needs to be immunized with the commercial pentavalent vaccine, the EV-A71 vaccine could be included in the Expanded Programme on Immunization Vaccines. However, interactions with the pentavalent vaccine will need to be evaluated.

There also remains insufficient information on the inactivated EV-A71 vaccine-induced immunity to ensure wide and safe use of the vaccine inside and outside of China. Samples collected during Phase III Clinical Trials should be analyzed for immune response types and immune correlates of protection [45]. As Coxsackie type A16 (CVA-16) is also a leading cause of severe HFMD infections, there should be more research into formulating an inactivated bivalent EV-A71-CA16 vaccine to effectively prevent major HFMD outbreaks, commonly caused by these two pathogens.

## 5. Development of Viral-Like Particles as EV-A71 Vacines

Recent research has also focused on virus-like particles (VLP) as good vaccine candidates. VLPs resemble the authentic virus in terms of morphology, capsid proteins and protein composition, but are devoid of genetic material. Hence, VLPs are not infectious but can self-assemble in eukaryotic expression systems such as *Saccharomyces cerevisiae* [28] and *Pichia pastoris* [46]. More importantly, VLPs contain an arrangement of epitopes on its surface that can elicit NtAb against that particular virus. Indeed, VLPs have been developed as licensed vaccines for human papillomavirus and Hepatitis B virus [47]. Therefore, there remains potential for VLPs to be an excellent choice of vaccine for EV-A71.

In a recent publication by Zhang *et al.* (2015) [48], high yield production of recombinant VLPs of EV-A71 (approximately 150 mgVLP/Liter of yeast culture) was achieved in *Pichia pastoris.* Maternal immunization with the VLP co-expressing P1 and 3CD proteins of EV-A71 was able to protect neonatal mice in both intraperitoneal and oral challenge against EV-A71. The transgenic *Pichia pastoris* produced more VLPs than that reported for baculovirus/insect cell expression system as the latter only produced 64.3 mg/L under optimized condition. The overall cost of insect cell culture is relatively high and there is a potential risk of contamination with baculovirus particles. Recombinant virus-like particles produced from baculovirus formulated with CFA/IFA adjuvants elicited a neutralization titer of 1/160 which was significantly lower than the neutralization titer (1/640) elicited by the inactivated EV-A71 formulated in alum [49].

Nevertheless, with advances in structural vaccinology, enhanced baculovirus/adenovirus VLPs can be constructed that could rival yeast VLPs. One such example was carried out by Lin *et al.*, (2015) [50] who constructed recombinant baculoviruses (BacF-P1-C3CD) that expressed P1 under the polyhedrin (*polh*) promoter and 3CD under the CMV promoter, dramatically improving VLP yield while alleviating VLP degradation. Infection of High Five (TM) cells with BacF-P1-C3CD enhanced the total and extracellular VLP yield to ≈268 and ≈171 mg/L, respectively. Similar to the approach used by Lin *et al.* (2015) [50], Tsou *et al.* (2015) [51] constructed a recombinant adenovirus (Ad-EVVLP) with the EV71 P1 and 3CD region inserted into the E1/E3-deleted adenoviral genome. Mouse immunogenicity studies showed that Ad-EVVLP-immunized antisera neutralized the B4 and C2 genotypes of EV71. In mouse challenge models, Ad-EVVLP also successfully induced anti-CV-A16 immunitiy. These results collectively show that enhanced baculovirus/adenovirus VLPs can enhance neutralizing antibody and protective cellular immune responses to prevent EV71 infection and potentially cellular immune responses against CV infection.

In addition, there is an increasing trend to develop a bivalent VLP vaccine against both EV-A71 and CV-A16 as both viruses tend to co-circulate in major HFMD outbreaks. As such, Zhao and colleagues (2015) [52] constructed a chimeric EV-A71 VLP (ChiEV-A71 VLP) that also contained a CV-A16-SP70 epitope. Immunization of mice with ChiEV-A71 VLPs conferred both Th1/Th2 dependent immune responses against EV-A71 and CV-A16. In addition, passive immunity in neonatal mice with anti-ChiEV-A71 VLPs sera elicited full protection against EV-A71 and CV-A16 infection. Furthermore, Xu *et al.* (2015) [53] constructed a bivalent chimeric VLP (HBc-E1/2) presenting the VP1 and VP2 epitopes of EV71 using hepatitis B virus core protein (HBc) as a carrier and discovered that it could cross-neutralize against several EV-A71 B and C sub-genotypes and CV-A16 strains of the B1b genotype and the G10 strain belonging to the A genotype, with titers ranging Ref No: 1 from 1:8 to 1:16. Further analysis of the EV-A71 crystal structures showed that the VP2 (aa141–155) epitope was close to the VP1 GH loop (residues 208–222), and it was exposed on the surface of EV-A71 and CV-A16. These results illustrate that the chimeric VLP HBc-E1/2 is a promising candidate for a broad-spectrum HFMD vaccine.

Lyu *et al.*, (2014) [54] discovered that inserted foreign peptides that are well exposed on viral particle surface did not cause significant structural changes on the capsid, viral replication and viral uncoating process. This would provide more insights into vaccine development against HFMD. In their most recent study, Lyu *et al.* (2015) [55] showed that the crystal structures of EV-A71 VLP and chimeric EV-A71/CV-A16 VLP contained major neutralization epitopes of EV-A71 that are mostly preserved in both VLPs. The replacement of 4 amino acid residues in the VP1 GH loop of the SP70 epitope was able to change the chimeric VLP to elicit neutralization responses against both EV-A71 and CAV16. The mutated VP1 GH loop in the chimeric VLP was well exposed on the particle surface and exhibited a surface charge potential different from that contributed by the original VP1 GH loop in EV-A71 VLP. The study provided insights and evidence that the yeast-produced VLPs can be developed into novel vaccines against HFMD and other enterovirus-associated diseases.

Using reverse genetics, Wang *et al.* (2013) [56] engineered an EV-A71 virus carrying biomimetic peptides that could control a calcium phosphate biomineralization process. The mineralization process can be biologically induced onto vaccine surfaces under physiological conditions, generating a mineral exterior that could improve thermal stability and physicochemical properties. Interestingly, the self-biomineralized vaccine could be stored at 26 °C for more than nine days and at 37 °C for approximately one week. Such a combination of genetic technology and biomineralization provides an economic solution for current vaccination programs, especially in developing countries that lack expensive refrigeration infrastructures. Improving the efficacy and thermostability of vaccines is desirable and has been highlighted as a Grand Challenge in Global Health by the Gates Foundation.

There has been a greater understanding of the crystal structure of the EV-A71 virus. In an earlier study, it was discovered that EV-A71 had a “pocket factor” that was partly exposed on the floor of the canyon. This “pocket factor” was demonstrated to interact with polar residues on the canyon floor [57]. In addition, Wang *et al.* (2012) [58] utilized structural analysis of mature and empty EV-A71 particles and discovered that empty particles were also found to contain a “pocket factor” in the EV-A71 capsid. Hence, this provided a model for enterovirus un-coating whereby the VP1 GH loop acted as an adaptor-sensor for cellular receptor attachment. With a greater understanding of the EV-A71 crystal structure, better vaccines can be constructed.

Interestingly, structural vaccinology is now emerging as a promising strategy in the rational design of vaccines. An understanding of the relationship between 3D crystal structures, biological function and immunological cross reactivity can lead to the design of more effective viral vaccines. Recent studies of the sequence-variable regions of the HIV-1 gp120 envelope glycoprotein have shown that there are conserved immunological and structural features in these regions. This is supported by data showing that sequence-variable loops of HIV-1 gp120 such as the V2 and V3 loops can give rise to a spectrum of antibodies, some with very narrow specificities for only one or a few strains of HIV-1, and others with broad immunological and neutralizing activities against diverse HIV-1 strains [59].

## 6. Conclusions

Vaccines against viral diseases could be potentially improved if the immunogenicity and thermal stability of the vaccine could be enhanced. Although three companies from China have completed pre-clinical studies to develop an inactivated vaccine, there needs to be post market evaluation of immunogenicity on whether the NtAb elicited by one EV-A71 sub-genotype could cross-neutralize other sub-genotypes or confer protection against serious HFMD. In addition, bivalent VLP vaccines against both EV-A71 and CV-A16 are being studied extensively, but it remains questionable if CV-A16 is the major Coxsackie virus causing HFMD, as 9% of cases are attributed to CV-A10, 8.3% to CVA-6, and only 6.9% of HFMD cases are attributed to CV-A16 in a 2010/2011 outbreak in Shanghai. It is possible that other VLP bivalent vaccines against EV-A71 and CV-A10 or EV-A71 and CV-A6 should also be taken into consideration. Rational design of EV-A71 vaccines should focus on the development of epitope-scaffold immunogens, targeting conserved structural features but incorporating neutralizing epitope(s) that can elicit broad, cross strain neutralizing antibodies. More importantly, a general strategy for vaccine improvement should be developed to assist in the execution and expansion of immunization programs globally, especially for the low- and middle-income countries where they are needed the most.
